# EXPAREL® (Long-Acting Liposomal Bupivacaine) Use for Popliteal Nerve Block in Postoperative Pain Control after Ankle Fracture Fixation

**DOI:** 10.1155/2020/5982567

**Published:** 2020-07-23

**Authors:** Patrick Discepola, Mohamed Bouhara, Minho Kwon, Bilal A. Siddiqui, Trevor A. Whitwell, Swetha Y. Sanghvi, Keith D. Cook, Ross E. Moore, Anna Korban, Jean D. Eloy

**Affiliations:** ^1^Anesthesiology, Rutgers New Jersey Medical School, Newark, NJ, USA; ^2^Podiatry, University Hospital, Newark, NJ, USA

## Abstract

EXPAREL® has been used successfully to prolong postoperative pain control when applied as a wound infiltrate. EXPAREL® has not yet been approved for use in regional anesthesia to prolong postoperative pain control. We conducted a clinical case series of 4 patients using EXPAREL® for sciatic blocks via the popliteal fossa approach. Our results suggested that there is a large degree of variability in response to the medication. These inconsistent results and the possibility of bimodal kinetics creating analgesic gaps as seen in two of our patients indicate that more studies with larger sample size are needed to better characterize these phenomena and determine if more consistent results can be obtained in a future clinical trial.

## 1. Introduction

Managing postoperative pain remains a paramount topic in anesthesiology, highlighted by the significant amount of patients who continue to report discomfort after inpatient and outpatient surgeries [1]. It is also well-known that lower limb orthopedic surgeries induce prolonged pain that, in turn, require increased use of multimodal analgesia in the postoperative period [2]. To alleviate this issue, the popliteal fossa nerve block, which targets the sciatic nerve, is often used to effectively control postoperative pain and lower opioid requirements for patients undergoing foot and ankle surgeries [3, 4]. Bupivacaine, a local anesthetic and analgesic, is currently used as an agent to address postsurgical analgesia. However, administering a large dose of bupivacaine for nerve blockade has been linked to complications such as infection, hematoma, vascular puncture, and severe systemic side effects [5–7]. EXPAREL® (multivesicular liposomal bupivacaine) is a multivesicular liposomal form (DepoFoam drug delivery systems) of encapsulated bupivacaine that allows for the slow diffusion of the drug over an extended period of time. Compared to bupivacaine, which only lasts approximately 8 hours, EXPAREL® lasts around 72 hours, almost a 9-fold difference. Thus far, EXPAREL® has been shown to provide successful prolonged analgesia after wound infiltration during several surgical procedures [8–10]. In addition to the long-lasting pain relief, studies also highlight the improved clinical outcomes of patients and lower economic costs associated with liposomal bupivacaine when compared to bupivacaine HCl [8]. However, studies evaluating the use of EXPAREL® for nerve blocks are limited, particularly in a surgical patient population. The objective of this case series was to evaluate the efficacy of the study drug EXPAREL® with respect to the safety and duration of the postoperative analgesic effect, specifically on four subjects following a single-dose injection of EXPAREL® via a popliteal nerve block and to help determine whether a randomized, double-blind prospective study is warranted.

The Food and Drug Administration (FDA) has not approved the use of EXPAREL® in regional anesthesia. We applied for and were granted an IND (#121369) Exemption to conduct a clinical case series of 4 patients using EXPAREL® for sciatic blocks via the popliteal fossa approach. An approval from our Institutional Review Board (IRB) was also obtained.

## 2. Materials and Methods

Four patients undergoing ORIF ankle surgery were selected using the following inclusion and exclusion criteria.

### 2.1. Inclusion Criteria

- Male or female; age: 18–65 years
- Undergoing ankle fracture open reduction and internal fixation (ORIF) or ankle fusion surgery
- American Society of Anesthesiologists (ASA) physical status classification I–III
- Subjects who are willing and able to provide consent
- Subjects who have a working telephone

### 2.2. Exclusion Criteria

- Non-English-speaking (none of the study team members are fluent in Spanish)
- BMI greater than 40 kg/m^2^ (greater risk associated with doing blocks on patients with a BMI > 40)
- Known allergy or contraindication to medications being used
- Known allergy to local anesthetics
- Subjects who are currently pregnant, nursing, or planning to become pregnant during the study or within 1 month after study drug administration
- History of drug abuse
- History of impaired renal function, rheumatoid arthritis, or peripheral neuropathy
- Subjects with no response to the Semmes Weinstein test

After study explanation and informed consent was obtained, each patient was enrolled in the study. Prior to surgery, each patient was taken to the Postoperative Anesthesia Care Unit (PACU) where standard American Society of Anesthesiologists (ASA) monitors were placed.

Each subject received a single dose of study drug EXPAREL® 1.3% 20 ml (266 mg) via ultrasound-guided popliteal nerve block by our regional anesthesiology team. Once the block was placed, the patient was, then, taken to the operating room for surgery. Intraoperative narcotic use was prohibited with the exception of (intravenous) fentanyl boluses during induction or need to treat an adverse event such as tourniquet pain. Use of alternate local anesthetics by both the anesthesiologist and surgeons was also prohibited. On arrival to the PACU, an assessment of block success was quantified by determining the presence of sensory or motor deficits in the targeted dermatomes. Neurological and sensory assessments were conducted on arrival, at 60 minutes and at 120 minutes. Pain scores and sedation scores were obtained using the Visual Analogue Scale (VAS) for pain and Ramsay Sedation Scale (RSS). The VAS is a verified, subjective measure for acute and chronic pain. Scores from 0 to 10 are recorded by making a handwritten mark on a 10 cm line that represents a continuum between “no pain” and “worst pain [11].” The RSS tests a patient's arousal by dividing the patient's level of sedation into six categories ranging from severe agitation to deep coma [12]. Both the VAS and RSS scores were obtained in intervals as per our PACU protocol of every 5 minutes for the first 15 minutes, 15 minute intervals for the next hour, and then every 30 minutes until discharge. After the stay in the PACU, Percocet (oxycodone 5 mg/325 mg acetaminophen) 1 or 2 tablets every 6 hours was prescribed by the surgical team for breakthrough pain. The doses and timing of all pain medications were recorded during hospitalization, and the patients were provided with a diary to record pain scores and medications taken at home. A pain questionnaire, which addressed the quality of postoperative pain relief, VAS pain scale, and incidence and severity of any side effects (including palpitations, nausea, vomiting, burning, numbness, tingling, and rebound), was completed by each patient daily at the end of postoperative day (POD) #1 through POD #3. On POD #3, the subjects were asked to give an overall opinion of their experience on a subjective 5-point scale, wherein a score of 5 equates to a completely satisfied experience.

## 3. Results

### 3.1. Case 1

The patient was a healthy 46-year-old female with no significant medical history (patient weight: 90.1 kg and height: 67 inches) who presented with a lateral malleolus fracture after a fall. She underwent an ORIF ankle, and surgery lasted about an hour and half. Her baseline preoperative pain score was 0, and she had no sensory or motor deficits. Preoperative nerve block placement was uneventful, and the patient received 20 mL of EXPAREL® 1.3% 20 ml (266 mg). On arrival to the PACU, she reported no cold sensation in the targeted dermatome areas; however, she reported 10/10 pain on VAS. In addition, the patient complained of pruritis, and she received 0.5 mg hydromorphone IV and diphenhydramine 50 mg IV. Her followed-up pain scores were 3/10 and 0/10 at 60 minutes and 120 minutes, respectively. On POD #1, the patient recorded a highest pain score of 8/10 and required 2 doses of Percocet 5/325 total for the day. On POD #2, the patient recorded a highest pain score of 10/10, so she took Advil for breakthrough pain as she reported pruritus after taking Percocet. On POD #3, her highest pain score was 4/10, and she continued to take Advil for breakthrough with two doses total for the day. The patient reported a 4/5 overall satisfaction score.

### 3.2. Case 2

This patient was a 48-year-old female with a history of diabetes mellitus (type 2) (patient weight: 95 kg and height: 64 inches) who presented with a bimalleolar ankle fracture after a fall. The patient underwent an ORIF ankle, and her surgery lasted one hour and 15 minutes. Her preoperative pain score was 0/10, and she had no sensory or motor deficits. Preoperative nerve block placement was uneventful, and the patient received a single dose of EXPAREL® 1.3% 20 ml (266 mg). On arrival to the PACU, she reported no cold sensation in the targeted dermatome areas, 0/10 pain on VAS, and had a Ramsay sedation score of 3/6. Her pain score remained 0/10 for the entire PACU stay, while her sedation score improved to 2/6. On POD #1, the patient recorded a highest pain score of 8/10; however, she took no pain medication. On POD #2 and #3, the patient recorded pain scores of 0/10 for both days and had no oral pain medication requirements. For overall satisfaction, that patient recorded 5/5 and reported that her block lasted about three and half days (approximately 84 hours).

### 3.3. Case 3

This patient was a 54-year-old female with no significant medical history (patient weight: 70.3 kg and patient height: 47 inches) who presented with a left bimalleolar fracture status after a fall. The patient underwent an ORIF ankle, and her surgery lasted 1 hour. Her preoperative pain score was 1/10, and she had no sensory or motor deficit. Nerve block placement was uneventful in the PACU where the patient received 20 mL of EXPAREL® 1.3% 20 ml (266 mg). The patient reported cold sensation in the targeted dermatome areas, with 3/10 pain. At 60 minutes of assessment, she had no cold sensation in the targeted dermatome and had a pain score of 5/10. At 120 minutes, her pain score was 0/10, and she did not require any pain medication while in the PACU. Postoperatively, the patient recorded 0/10 pain scores for POD #1–3 and took no breakthrough pain medication. She recorded an overall satisfaction score of 5/5 and reported that the block lasted about 72 hours.

### 3.4. Case 4

The subject was a 49 year-old female with a history of hypertension, depression, and chronic low back pain (patient weight: 104 kg and height: 68 inches) who presented with a right bimalleolar fracture status after a fall. The patient underwent an ORIF ankle, and her surgery lasted 1 hours and 20 minutes. Her home medications included hydralazine-hydrochlorothiazide, metoprolol, and Percocet. Her preoperative pain score was 8/10, and she had no sensory or motor deficits. Nerve block placement was uneventful, and the patient received 20 mL of EXPAREL® 1.3% 20 ml (266 mg). In the PACU, she was sedated, not oriented, and had a Ramsay sedation score of 3. At 60 minutes, her sedation score had declined to 2/6, and she reported no cold sensation in the targeted dermatome with a pain score 0/10. At 120 minutes, her sensory and pain status were unchanged. On POD #1, the patient recorded 2/10 pain scores and took 5 doses of Percocet 5/325 for breakthrough pain. The record kept by the patient reported that the block wore off about 12 hours after placement. She was evaluated in the outpatient podiatry clinic and confirmed to have no sensory or motor deficits. When the patient woke up on the morning of POD #2, she reported a return of numbness to her foot and was unable to move her toes. Despite the return of numbness, the patient recorded a highest pain score of 10/10 and took 7 doses of Percocet and three doses of Motrin 600 mg throughout the day. On POD #3, the patient recorded a highest pain score of 4/10 and took three doses of Percocet for breakthrough pain. She reported that numbness continued up to the level of her calf. She recorded an overall satisfaction score of 4/5. After POD #3, the numbness in the foot resolved except a portion of her lateral foot in the sural nerve distribution. In this area, she had continuous numbness and developed hyperesthesia that lasted for 3 months (Tables 1–3).

## 4. Discussion

Currently, there is no established method to consistently prolong a peripheral nerve block for greater than 24 hours other than placement of a perineural catheter [13]. Liposomal bupivacaine (EXPAREL®) has a theoretical potential of extending a single shot nerve block greater than 24 hours without catheter placement; however, data on this subject is still limited. Currently, this medication has shown some promising results in improving postoperative pain when administered as wound infiltration [8–10]. A retrospective study performed by Hutchins et al. evaluated the use of EXPAREL® in Transverse Abdominis Plane (TAP) blocks for abdominal hysterectomy patients. The study demonstrated decreased pain scores, postoperative nausea and vomiting, and shorter hospital stays in patients who received a TAP block with EXPAREL® when compared to patients who did not receive a TAP block [14].

Data regarding the use of EXPAREL® in the regional and neuraxial anesthetic technique are still very limited and have been evaluated in healthy volunteers. A randomized double-blind trial, conducted by Viscusi et al., on 30 healthy volunteers studied the effects of the liposomal bupivacaine versus standard bupivacaine HCl when administered in the epidural space. This study showed a significant difference in both degree and duration of sensory blockade while showing no significant difference in motor blockade [15]. A dose response study by Ilfeld et al. evaluated varying doses of liposomal bupivacaine in bilateral femoral nerve blocks in nonsurgical volunteers. Their data suggest an association with dose and degree of sensory block; however, there was much interindividual variability. Their study also showed a seemingly paradoxical inverse relationship between the motor block and medication dosage [16]. This inconsistency and variability appear to be reflected in our case series as well. Of our 4 patients, only 2 had significant pain relief that lasted through the perioperative period. The patient in case #1 had significantly elevated pain scores in both the PACU and the postoperative days, requiring a significant amount of oral breakthrough pain medications. Although the patient endorsed some loss of cold sensation in the targeted dermatome, the high pain scores and breakthrough requirements seem to indicate a failed block not related to the study medication. The patient in case #2 had pain scores of 0/10 ad endorsed loss of cold sensation in the targeted dermatome indicating a successful block. The following day, she documented a VAS score 8/10, but the she did not take any breakthrough pain medication. On POD #2 and #3, she had VAS scores of 0/10 and continued to document numbness. The patient endorsed the numbness lasted approximately 84 hours. The return of pain on POD #1 followed by returning to a pain-free state (VAS 0/10) without oral breakthrough requirement on POD #2 and #3 is possibly the result of bimodal pharmacokinetics of EXPAREL®. The study by Hu et al. analyzed data from four different randomized trials involving wound infiltration of surgical wounds with EXPAREL® to determine the pharmacokinetic profile of liposomal bupivacaine versus standard bupivacaine. Their analysis showed that EXPAREL® appears to have a bimodal pharmacokinetics. In terms of plasma concentration, an initial peak occurred similar to standard bupivacaine HCl; however, there was a second plasma peak that occurred between 10 and 36 hours after injection [17]. This bimodal model of peak concentrations is thought to be, likely, a reflection of the 3% nonliposomal bupivacaine added to EXPAREL® to improve onset time, as well as the delayed release from the liposomes themselves.

The patient in case #3 had a successful postoperative course, having VAS scores of 0/10 both postoperatively and the subsequent three days. She reported that her numbness lasted about 72 total hours after the block placement. She required no oral breakthrough analgesics during the study period. In case #4, the patient's PACU course was indicative of a successful block with VAS scores of 0/10 and no oral analgesic requirements. Later in the evening, about 10 hours after block placement, the patient documented the return of sensation and pain requiring multiple breakthrough doses of Percocet. Her examination on POD #1 confirmed a return of sensation and had no motor deficits; however, on a subsequent follow-up phone conversation on POD #2, the patient indicated a return of numbness in a sciatic distribution and difficulty moving her toes. This was the second patient in our case series to demonstrate the return of postoperative pain followed by a return of numbness. This numbness persisted through POD #3; however, as this began to resolve, the patient began developing hyperesthesia and continued numbness in the sural nerve distribution that continued for 3 months. It is unclear whether these continued symptoms were the result of the study medication or nerve damage during block placement or direct trauma to the nerve itself intraoperatively. We hypothesize that the hyperesthesia was not the result of the EXPAREL® itself as a study by Richards et al. evaluated the potential neurotoxicity of EXPAREL® on the brachial plexus of rabbits and dogs. The study showed only minimal to mild granulomatous inflammation of adipose in doses of 30 mg/kg EXPAREL® when compared to bupivacaine and saline. There was no evidence of neural tissue injury in this study even at high doses [18]. The results of our case series are consistent with safe use of EXPAREL®, including the safety concerns surrounding local anesthetic toxicity. All four of our patients report no adverse effects of the block indicative of local anesthetic toxicity.

## 5. Conclusions

Liposomal bupivacaine (EXPAREL®) has shown to be effective at prolonging postoperative pain control when administered by wound infiltration; however, the effectiveness of this medication in use through nerve blocks is yet to be established. The results of this case series of EXPAREL® use in sciatic nerve block appear to be similar to those of previous studies conducted on healthy volunteers suggesting that there is a large degree of variability in response to the medication. These inconsistent results and the possibility of bimodal kinetics creating analgesic gaps as seen in two of our patients indicate that more studies with larger sample size are needed to better characterize these phenomena and determine if more consistent results can be obtained in a future clinical trial.

## Figures and Tables

**Table 1:** Patient pain/sedation score and breakthrough requirements in the PACU.

Patient	Time	Pain score	Pain medication	RSS
Case #1	Arrival	10	Dilaudid 0.5 mg IV	1
	60 minutes	3	None	2
	120 minutes	0	None	2

Case #2	Arrival	0	None	3
	60 minutes	0	None	2
	120 minutes	0	None	2

Case #3	Arrival	3	None	2
	60 minutes	5	None	3
	120 minutes	0	None	2

Case #4	Arrival	0	0	3
	60 minutes	0	0	2
	120 minutes	0	0	2

**Table 2:** Patient pain score and breakthrough requirements in postoperative days #1–3.

Patient	Postop. day	Pain score	Daily Percocet consumption	Daily ibuprofen consumption
Case #1	1	8	2 tablets	0
	2	10	0	2400 mg
	3	4	0	800 mg

Case #2	1	8	0	0
	2	0	0	0
	3	0	0	0

Case #3	1	0	0	0
	2	0	0	0
	3	0	0	0

Case #4	1	20	5 tablets	0
	2	10	7 tablets	1800 mg
	3	4	3 tablets	0

**Table 3:** Block duration by the patient in hours.

Patient	Block duration (hours)
Case #1	Indeterminate
Case #2	84
Case #3	72
Case #4	12

## Data Availability

The case data used to support the findings of this study are included within the article.
